# Continuous Flow Synthesis of High Valuable N-Heterocycles via Catalytic Conversion of Levulinic Acid

**DOI:** 10.3389/fchem.2019.00103

**Published:** 2019-02-26

**Authors:** Daily Rodríguez-Padrón, Alain R. Puente-Santiago, Alina M. Balu, Mario J. Muñoz-Batista, Rafael Luque

**Affiliations:** ^1^Grupo FQM-383, Departamento de Química Orgánica, Universidad de Cordoba, Cordoba, Spain; ^2^Scientific Center for Molecular Design and Synthesis of Innovative Compounds for the Medical Industry, People's Friendship University of Russia (RUDN University), Moscow, Russia

**Keywords:** N-heterocycles, heterogeneous catalysis, graphitic carbon nitride, continuous flow, platinum, Levulinic acid

## Abstract

Graphitic carbon nitride (g-C_3_N_4_) was successfully functionalized with a low platinum loading to give rise to an effective and stable catalytic material. The synthesized g-C_3_N_4_/Pt was fully characterized by XRD, N_2_ physisorption, XPS, SEM-Mapping, and TEM techniques. Remarkably, XPS analysis revealed that Pt was in a dominant metallic state. In addition, XPS together with XRD and N_2_ physisorption measurements indicated that the g-C_3_N_4_ preserves its native structure after the platinum deposition process. g-C_3_N_4_/Pt was applied to the catalytic conversion of levulinic acid to N-heterocycles under continuous flow conditions. Reaction parameters (temperature, pressure, and concentration of levulinic acid) were studied using 3 levels for each parameter, and the best conditions were employed for the analysis of the catalyst's stability. The catalytic system displayed high selectivity to 1-ethyl-5-methylpyrrolidin-2-one and outstanding stability after 3 h of reaction.

## Introduction

Biomass has emerged as a competitive alternative for the generation of highly sustainable fuels, chemicals, and drugs (Tuck et al., 2012; Sankaranarayanapillai et al., 2015; Ruppert et al., 2016; Hu et al., 2017; Tang et al., 2017; Filiciotto et al., 2018; Kucherov et al., 2018; Xu W. et al., 2018). A useful strategy for converting biomass feedstocks into fuels and chemicals is based on the transformation of platform molecules, which exhibit high functionality, to form added-value compounds (Serrano-Ruiz et al., 2011; Verma et al., 2017). In this direction, levulinic acid (LA) is a well-known platform molecule that has been widely used toward the fabrication of several valuable compounds such as γ-valerolactone (GVL), which represent a promising fuel source, levulinate esters, which are viable additives for gasoline and diesel transportation fuels, and pyrrolidones, which are involved in industry as surfactants, intermediates for pharmaceuticals, dispersants in fuel additive compositions, solvents and agrochemicals (Huang et al., 2011; Bermudez et al., 2013; Colmenares and Luque, 2014; Touchy et al., 2014; Chatzidimitriou and Bond, 2015; Yan et al., 2015; Ruppert et al., 2016; Gao et al., 2017; Sun et al., 2017; Xu C. et al., 2018).

In the last years, the use of heterogeneous catalysts for the valorization of LA into useful compounds, especially pyrrolidones, has been widely applied (Du et al., 2011; Ogiwara et al., 2016). For instance, the reductive amination of LA with amines in liquid phase has been described using precious metals such as Au, Pd, Pt, Ru, In, and Ir, supported on carbon or metal oxides owing to their large portfolio of versatile applications (Du et al., 2011; Chatzidimitriou and Bond, 2015; Ogiwara et al., 2016; Zhang et al., 2017). Additionally, a number of endeavors have been made to synthesize novel materials with desirable catalytic properties in order to improve the efficiency of the LA catalytic upgrading toward the production of pyrrolidones (Gao et al., 2017; Sun et al., 2017; Wu et al., 2017). Ultimately, innovative advancements on the design of active and stable heterogeneous catalysts composed of carbon-based materials have been proposed. Zheming Sun et al. have reported a new class of solid molecular N-heterocyclic carbon (NHC) catalysts for the solvent-free reductive amination of biomass-derived levulinic acid to obtain a large variety of interesting structural configurations of *N*-substituted pyrrolidones (Sun et al., 2017). In this regard, NHC-Ru polymer showed high catalytic performance and remarkable reusability, allowing the development of one-pot tandem reductive reactions of LA with aldehydes or ketones.

Graphitic carbon nitride, generally known as g-C_3_N_4_, is recognized as the most stable allotrope among various carbon nitrides under ambient conditions. The surface chemistry of its polymeric structure can be easily controlled via molecular-level modification and surface engineering. Additionally, the polymeric nature of g-C_3_N_4_ guarantees sufficient flexibility of the structure, which can serve as a compatible matrix for the anchorage of various inorganic nanoparticles and consequently can be successfully applied in a myriad of photocatalytic applications (Muñoz-Batista et al., 2015b, 2016, 2018; Xue et al., 2015; Fontelles-Carceller et al., 2016; Sastre et al., 2016; Zeng et al., 2017; Hak et al., 2018; Majeed et al., 2018). Despite the mentioned applications, graphitic carbon nitride-based materials have not been broadly employed toward the catalytic valorization of biomass-derived chemicals. A representative example in which an organic sulfonated graphitic carbon nitride was used for conversion of carbohydrates into furanics and related value-added products can be highlighted (Verma et al., 2017).

We report herein the reductive amination of levulinic acid into highly valuable pyrrolidones driven by g-C_3_N_4_/Pt composites as a competitive catalyst. The catalytic processes were performed under flow conditions which ensure high control over reaction conditions, fast and effective reagent mixing and shorter times of reactions (Bermudez et al., 2013; Chen et al., 2015; Gemoets et al., 2016; Muñoz-Batista et al., 2018).

## Experimental

### Materials

All chemicals were obtained from Sigma–Aldrich with pure analytical degree.

### Synthesis of g-C_3_N_4_/Pt Composites

The graphitic carbon nitride was obtained by calcination of melamine in a semi-closed system at 580°C for 4 h using a heating rate of 5°C min^−1^. In order to improve its superficial area, the obtained bulk g-C_3_N_4_ was treated by ultrasonication for 5 h in deionized water using 1 mg mL^−1^. The platinum component was deposited using a simple chemical reduction method. The g-C_3_N_4_ support was suspended by stirring in deionized water solution for 30 min. Then, the proper quantity of H_2_PtCl_6_ was added to the solution to get 1 wt.% of Pt on metal basis and kept under stirring for 15 min. Finally, a hydrazine aqueous solution was quickly added, where the molar ratio between Pt and hydrazine was fixed to 1:5. The resulting mixture was stirred for 30 min and separated by filtration. The separated solid was rinsed with distilled water and dried at 80°C for 16 h. The Pt loading in the sample was 1 wt.%, confirmed by ICP-MS analysis in an Elan DRC-e (PerkinElmer SCIEX) spectrometer.

### Catalyst Characterization

XRD experiments were performed in the Bruker D8 Advance Diffractometer with the LynxEye detector. The XRD patterns were recorded in a 2θ scan range from 10 to 80°. Phase identification was carried out using Bruker Diffrac-plus Eva software, supported by the Power Diffraction File Database. N_2_ physisorption experiments were accomplished with the Micromeritics ASAP 2000 instrument. The sample was previously degassed for 24 h under vacuum (*p* < 10^−2^ Pa). Moreover, TEM images were recorded in the JEOL JEM 1400 instrument and assembled with a charge-coupling camera device. Samples were previously suspended in ethanol and deposited on a copper grid. SEM-EDX micrographs were acquired in the JEOL-SEM JSM-7800 LV scanning microscope. XPS measurements were accomplished with an ultrahigh vacuum multipurpose surface analysis instrument, SpecsTM. Prior to the analysis, the sample was evacuated overnight under vacuum (10^−6^ Torr). XPS spectra were acquired at room temperature using a conventional X-ray source with a Phoibos 150-MCD energy detector. XPS CASA software was employed to analyze the obtained results.

### Catalytic Experiments

Catalytic performance of the obtained catalytic materials was evaluated in the H-Cube Mini Plus™ flow hydrogenation reactor. The catalysts were packed (ca. 0.1 g of catalyst per cartridge) in 30 mm-long ThalesNano CatCarts. The system was firstly washed with (1) methanol and (2) acetonitrile (0.3 mL/min, 20 min for each solvent). A solution of levulinic acid in acetonitrile was subsequently pumped through and the reaction conditions were set. The required hydrogen was generated *in situ* during the reaction by water electrolysis in the H-Cube equipment. The reactions were followed for 120 min, where a stationary situation was reached, and the collected samples were analyzed by GC-MS.

The conversion, selectivity and stability achieved for the catalyst in the reaction were investigated by gas chromatography (GC) in an Agilent 6890N gas chromatograph (60 mL min^−1^ N_2_ carrier flow, 20 psi column top head pressure) using a flame ionization detector (FID). The capillary column HP-5 (30 m × 0.32 mm × 0.25 mm) was employed. In addition, the collected liquid fractions were analyzed by GC-MS—using the Agilent 7820A GC/5977B High Efficiency Source (HES) MSD—in order to identify the obtained products.

## Results and Discussion

**Figure 1:**
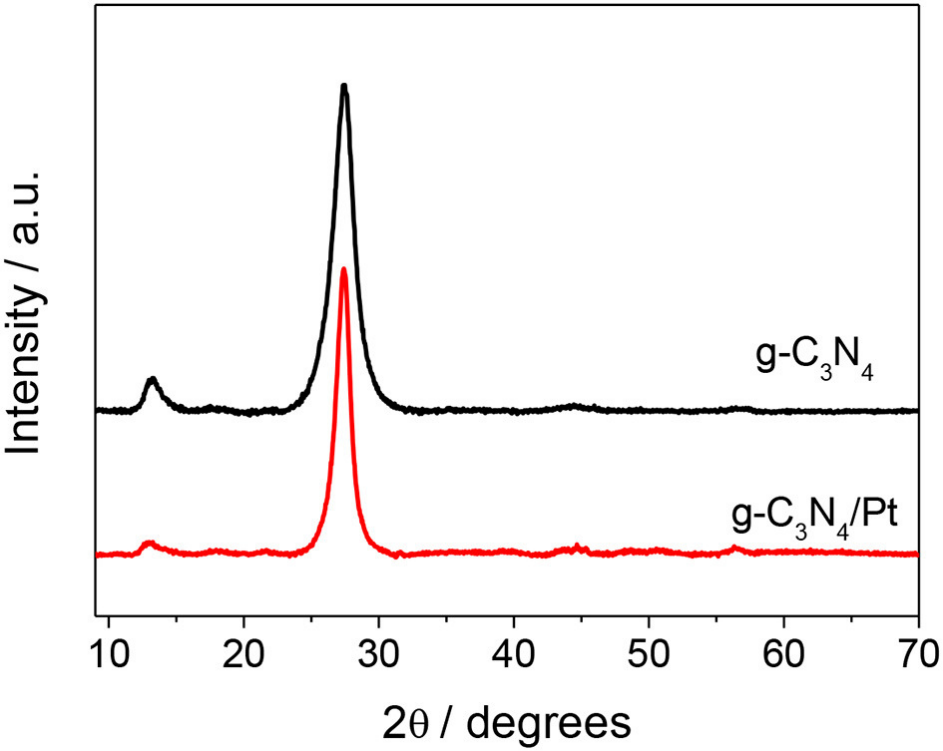
XRD patterns of g-C_3_N_4_/Pt sample and g-C_3_N_4_ reference.

X-ray diffraction analysis was employed to identify the structure and arrangement of the synthesized graphitic carbon nitride as well as the platinum-modified sample. As shown in Figure 1, both samples presented the typical interlayer-stacking (002) reflection of disordered carbon in a graphitic g-C_3_N_4_ layered structure and a peak around 13.1°, associated to the (100) reflection (Muñoz-Batista et al., 2016). The position of the (002) plane also showed the typical shift (~0.3), in comparison with the bulk counterpart, which can be related to the decrease of the interlayer distance, which takes place during the ultra-sonication process (Muñoz-Batista et al., 2017). As has been analyzed in previous reports, the limitation of the number of sheets stacked produces the weakening of interlayer forces with effect in the corrugation of the layers and the subsequent decrease of the corresponding distance between layers (Niu et al., 2012; Muñoz-Batista et al., 2017). The XRD pattern of g-C_3_N_4_/Pt has not displayed considerable changes in comparison with the unmodified material and therefore did not offer information about the Pt phases, most likely due to the relatively limited amount of the noble metal in the final material.

**Figure 2:**
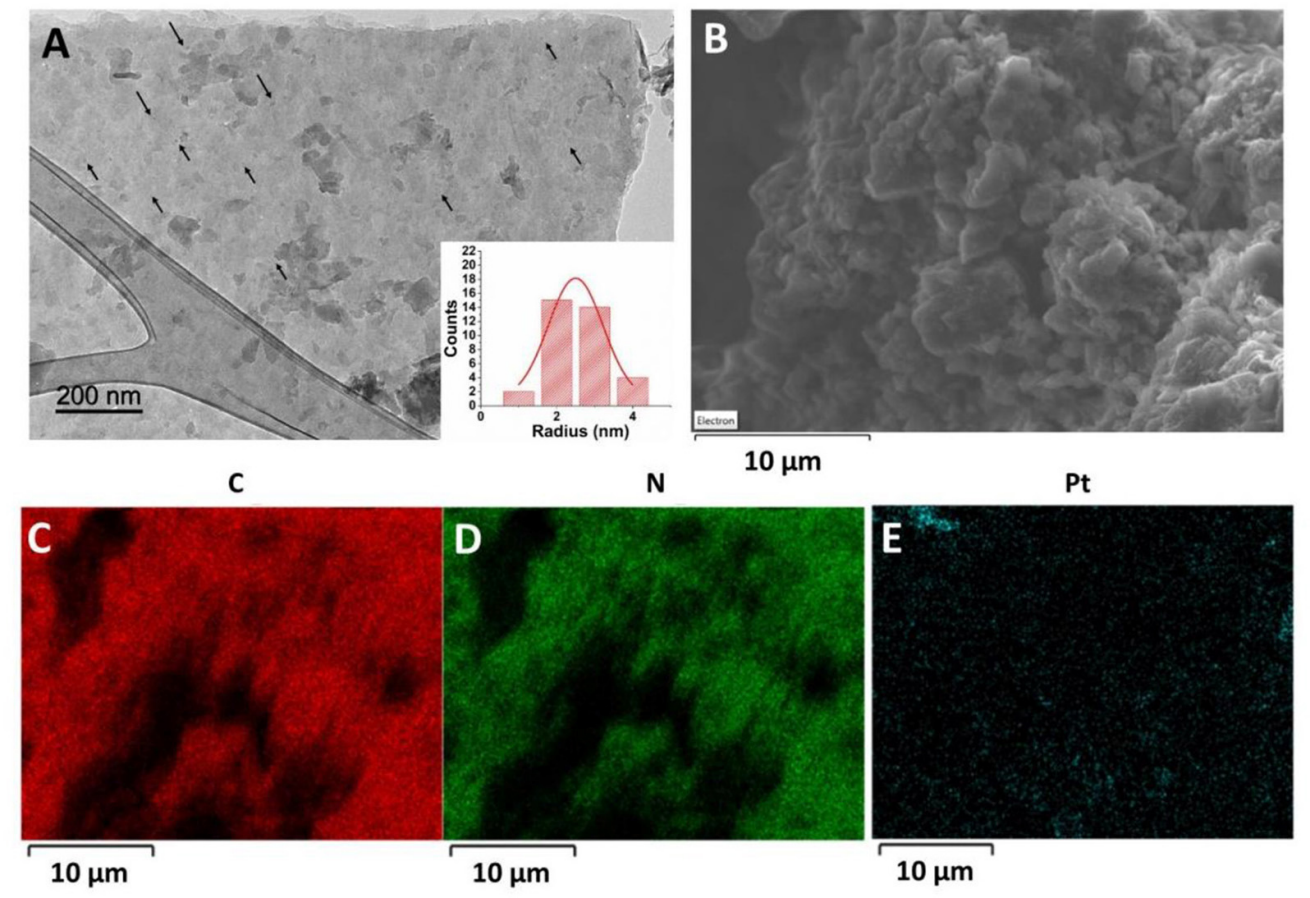
**(A)** TEM image of g-C_3_N_4_/Pt (Inset: size distribution of Pt nanoparticles), **(B)** SEM image of g-C_3_N_4_/Pt, SEM-mapping micrograph of g-C_3_N_4_/Pt for **(C)** carbon, **(D)** nitrogen, and **(E)** platinum.

Morphology of the synthetized g-C_3_N_4_/Pt was studied by microscopy (TEM and SEM) analyses (Figure 2). g-C_3_N_4_/Pt exhibited a laminar structure, as can be observed in Figure 2A. TEM analyses also allowed the identification of small platinum nanoparticles with a mean diameter of 2.5 nm on the g-C_3_N_4_/Pt surface (Table 1). Importantly, EDX-mapping micrographs of g-C_3_N_4_/Pt also confirmed the successful functionalization of graphitic carbon nitride with platinum, which depicts a relative homogeneous distribution. As is summarized in Table 1, the Pt-modified material showed rather similar values to the pure g-C_3_N_4_ reference in all the parameters determined by N_2_ physisorption (BET Surface area, pore volume, pore size). BET surface area above 50 m^2^ g^−1^ and a dominant mesoporous structure could be originated from the void volume created by the agglomeration of the g-C_3_N_4_ sheets, allowing an efficient deposition of the metallic entities (Table 1).

**Table 1:** Morphological properties of g-C_3_N_4_/Pt sample and g-C_3_N_4_ reference.

**Sample**	**BET surface area (m^**2**^ g^**−1**^)**	**Pore volume (cm^**3**^ g^**−1**^)**	**Pore size (nm)**	**Pt particle size (nm)**
g-C_3_N_4_	58	0.2	15.7	–
g-C_3_N_4_/Pt	54	0.2	15.8	2.5

**Figure 3:**
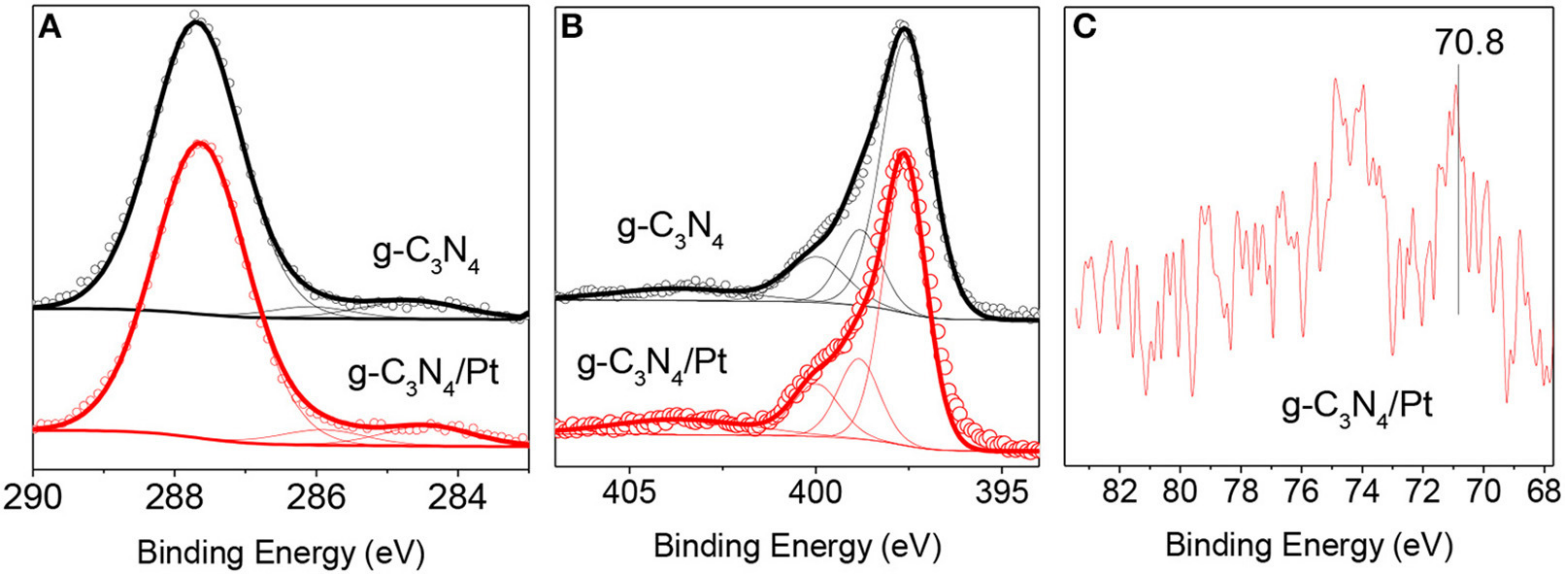
High-resolution XPS spectra of **(A)** C1s, **(B)** N1s, and **(C)** Pt4f in g-C_3_N_4_/Pt sample and g-C_3_N_4_ reference.

The structural analysis of g-C_3_N_4_/Pt and g-C_3_N_4_ was completed with the help of X-ray photoelectron spectroscopy. XPS measurements were carried out in order to provide information related to the carbon, nitrogen, and platinum components. Figure 3 shows the XPS spectra for the two catalytic systems, including C1s (Figure 3A), N1s (Figure 3B), and Pt4f (Figure 3C) regions. The summary of the N- and C-containing species contributing to the C1s and N1s peaks of the sample and g-C_3_N_4_ reference is presented in Table 2. The C1s XPS region showed contributions from C_3_-N (~286.2 eV), N-C-N (~287.6 eV) and C-C (~284.6 eV) (Muñoz-Batista et al., 2015a). C_3_-N and N-C-N can be exclusively ascribable to g-C_3_N_4_ while C-C contribution, which was also used as reference, energy can be associated with surface residues or defects in the nanopolymer structure (Wanger et al., 1979). For the N1s XPS region, besides C_3_-N (~399.1 eV) and N-C-N (~397.5 eV), two more contributions were used during the deconvolution procedure; N-H (~400.4 eV) and the typical broad pi-exc (~403.5 eV) (Muñoz-Batista et al., 2015a). In conclusion, Figures 3A,B as well as the data of Table 2 provide evidence of the strong similitude detected between the pure g-C_3_N_4_ and Pt/g-C_3_N_4_ samples. XPS also allowed the detection of minority components in the structure, namely Pt nanoparticles. Although the signal-to-noise ratio of the Pt XPS region (Figure 3C) is relatively low, the shape of the Pt4f is indicative of a dominant metallic state (~71 eV) (Fontelles-Carceller et al., 2017).

**Table 2:** C1s and N1s XPS region fitting results of g-C_3_N_4_ and g-C_3_N_4_/Pt.

N1s								
**Sample**	**C-N-C**	**%**	**(C)3-N**	**%**	**N-H**	**%**	**Pi-exc**.	**%**
g-C_3_N_4_	397.5	58.5	399.2	24	400.5	12	403.4	5.5
g-C_3_N_4_/Pt	397.2	58	399.0	24	400.4	12.5	403.6	5.5
**C1s**								
**Sample**	**C-C**	**%**	**(C)3-N**	**%**	**C-N-C**	**%**		
g-C_3_N_4_	284.6	8	286.2	5	287.6	87		
g-C_3_N_4_/Pt	284.6	9	286.2	5.5	287.5	85.5		

**Scheme 1:**
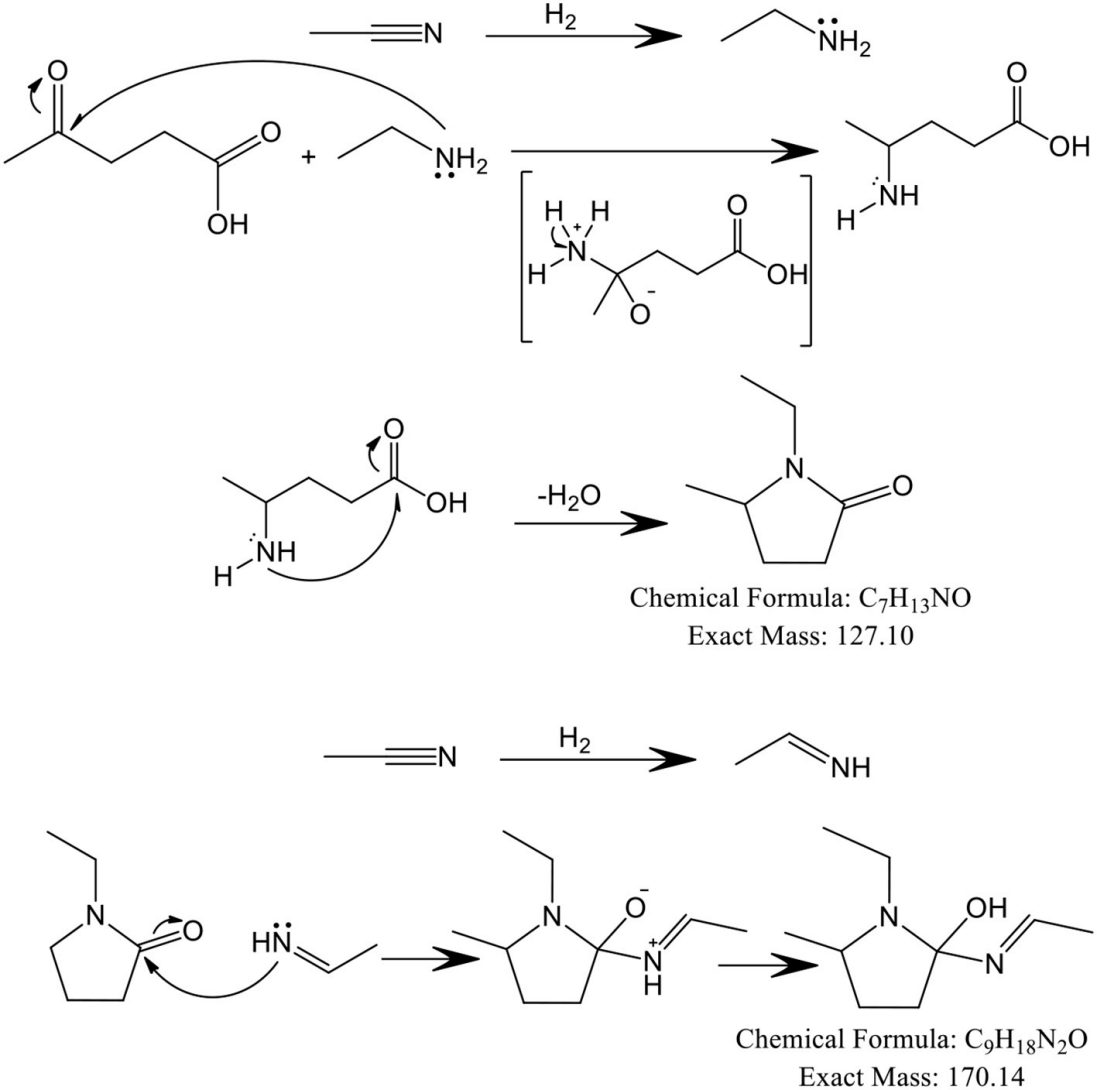
Proposed mechanism for the conversion of levulinic acid to N-heterocycles.

The catalytic performance of the prepared materials was evaluated in the conversion of levulinic acid to nitrogen-heterocycles under continuous flow conditions. N-heterocycles were obtained via condensation of levulinic acid, an 1,4-dicarbonyl compound, with an excess of ethylamine (Scheme 1) (Li, 2014). In this case, acetonitrile acts both as solvent and reactant, giving rise to ethylamine by *in situ* hydrogenation. The cyclization step involves a nucleophilic addition on a carbonyl group by the nitrogen of an intermediate. Levulinic acid acts as an electrophile both in the initial step of the reaction with the amine and in the cyclization step. After formation of the cyclic compound, the reaction proceeded via alcohol dehydration to produce the corresponding alkene. The hydroxyl (OH) group donates two electrons to H^+^, generating an alkylloxonium ion, which can act as a good leaving group. The formed alkene is effectively hydrogenated under hydrogen pressure to give rise to 1-ethyl-5-methylpyrrolidin-2-one, C_7_H_13_NO (127.10 g/mol).

In addition, the formation of 1-ethyl-2-(ethylideneamino)-5-methylpyrrolidin-2-ol, C_9_H_18_N_2_O (170.14 g/mol) can be understood if we take into account that hydrogenation of acetonitrile did not just give rise to ethylamine but also to ethanimine, which can further attack the carbonyl group of 1-ethyl-5-methylpyrrolidin-2-one with the consequential formation of 1-ethyl-2-(ethylideneamino)-5-methylpyrrolidin-2-ol.

**Figure 4:**
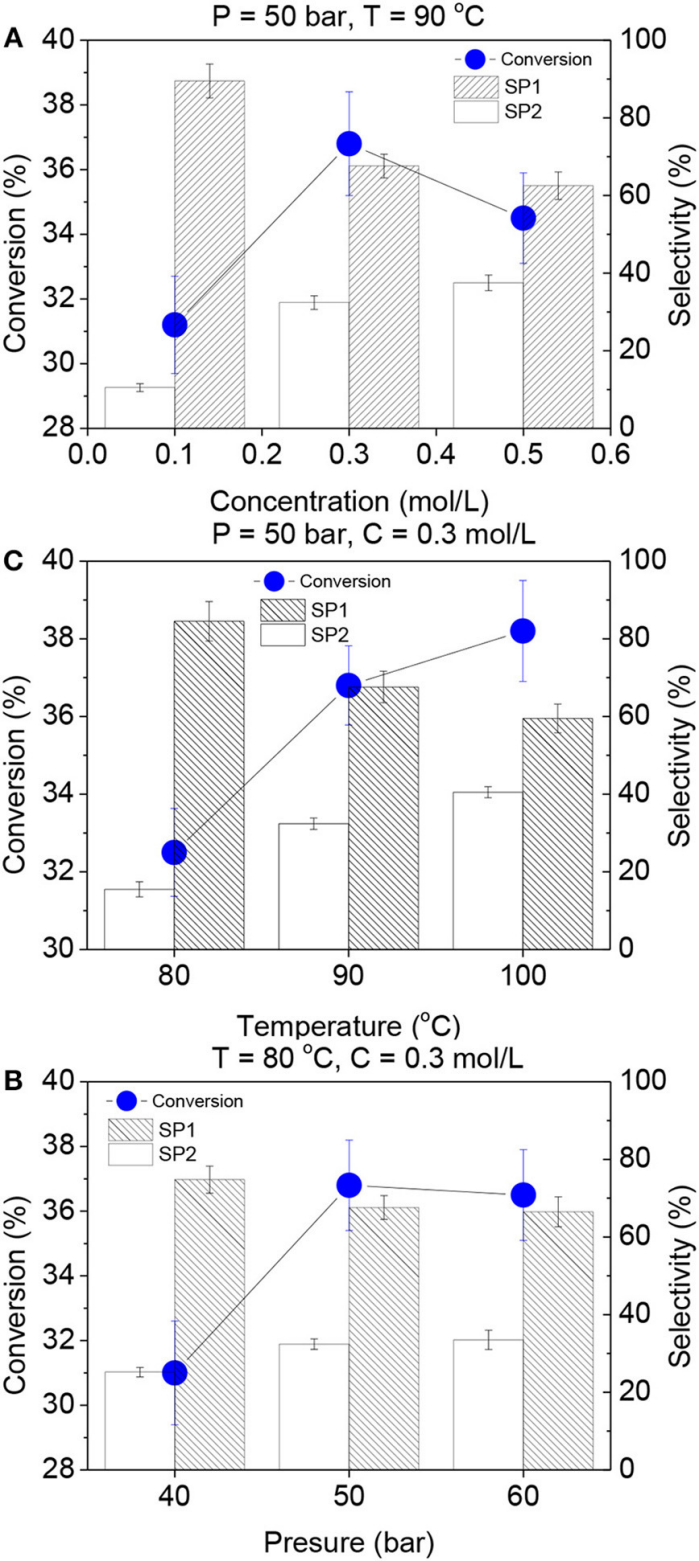
**(A)** Catalytic performance of g-C_3_N_4_/Pt at different concentrations of levulinic acid, **(B)** catalytic performance of g-C_3_N_4_/Pt at different temperatures, **(C)** catalytic performance of g-C_3_N_4_/Pt at different pressures. SP1: selectivity to 1-ethyl-5-methylpyrrolidin-2-one, SP2: selectivity to 1-ethyl-2-(ethylideneamino)-5-methylpyrrolidin-2-ol.

Firstly, a complete parametric analysis was accomplished in order to optimize the reaction conditions. In this regard, three levels of temperature, pressure and concentration were explored in the reaction of levulinic acid to N-heterocycles for 120 min (Figure 4). Carbon balance was achieved above 97% in all catalytic tests. Influence of the concentration in conversion and selectivity values was unraveled, as shown in Figure 4A. 0.3 mol/L was selected as the optimum concentration for the employed catalytic system. Although a lower concentration (0.1 mol/L) resulted in higher selectivity to 1 ethyl-5-methylpyrrolidin-2-one and the 0.5 mol/L concentration showed similar selectivity values, 0.3 mol/L gave rise to the best balance between conversion and selectivity.

The effect of temperature in the catalytic performance has been investigated by performing the reaction at 80, 90, and 100°C (Figure 4B). Additionally, influence of the system pressure was evaluated by accomplishing the reaction at 40, 50, and 60 bars (Figure 4C). In both cases an increment of the conversion and a decrease of the selectivity (1-ethyl-5-methylpyrrolidin-2-one) was observed for higher pressure and temperature values. Although 60 bars and 100° C conditions showed similar catalytic performance, 50 bars and 90° C were selected as the optimum pressure and temperature due to the good balance in conversion and selectivity, avoiding higher energy consumption reaction parameters. In addition, blank measurements were performed without a catalyst or employing g-C_3_N_4_, revealing that the reaction does not proceed in absence of an effective catalytic system.

**Figure 5:**
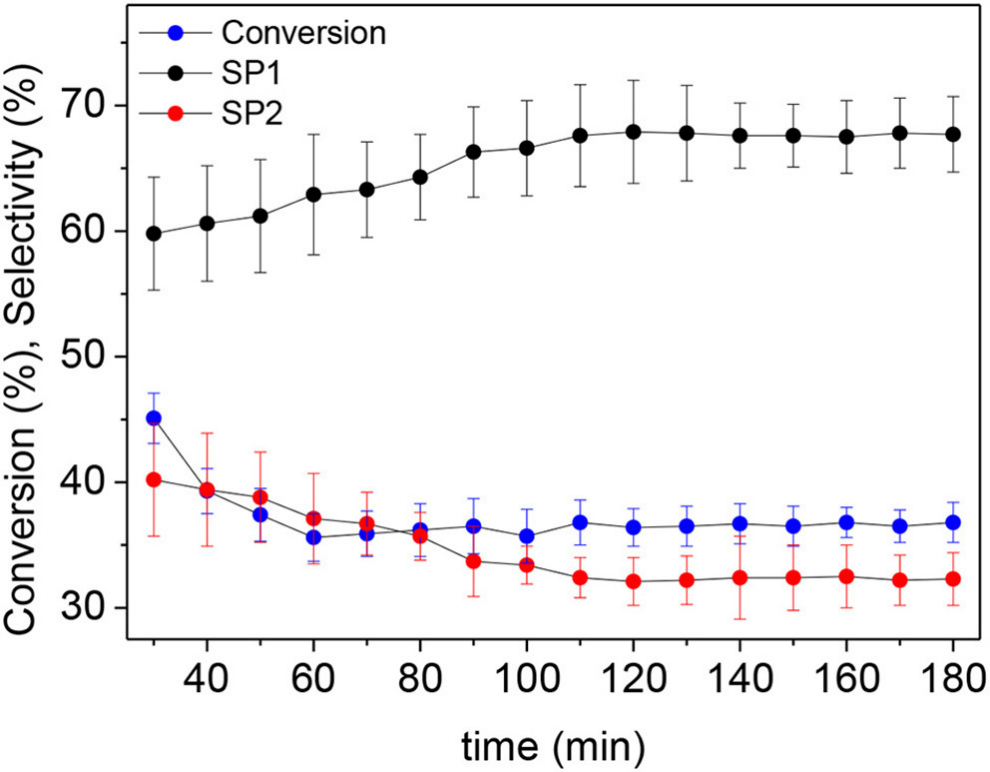
Catalytic performance of g-C_3_N_4_/Pt nanocatalyst during 180 min of reaction. Reaction conditions: 0.3 M levulinic acid solution in acetonitrile, 0.1 g of catalyst, T = 90 °C, P = 50 bar, Flow = 0.3 mL/min. SP1: selectivity to 1-ethyl-5-methylpyrrolidin-2-one, SP2: selectivity to 1-ethyl-2-(ethylideneamino)-5-methylpyrrolidin-2-ol.

Once the reaction parameters were optimized, the stability of the prepared catalytic system was investigated by performing the reaction for 3 h. After obtaining a stationary state (typically obtained after 120 min of reaction), a conversion of 36.8% employing g-C_3_N_4_/Pt was achieved. Figure 5 shows details of the flow catalytic process in terms of conversion and selectivity. The performed reaction gave rise to 1-ethyl-2-(ethylideneamino)-5-methylpyrrolidin-2-ol, C_9_H_18_N_2_O (170.14 g/mol), and 1-ethyl-5-methylpyrrolidin-2-one, C_7_H_13_NO (127.10 g/mol), with the last being the major product (67.6% of selectivity). Remarkably, the catalytic system displayed outstanding stability without considerable loss of activity for up to 3 h of reaction.

The structure of both products was proposed considering the fragmentation pattern in the MS spectra (Figure S1). The aforementioned compounds have a common fragmentation pattern and therefore a common skeleton. The molecular ions of C_7_H_13_NO and C_9_H_18_N_2_O were found at m/z 127.1 and 169.1, respectively.

## Conclusions

In summary, this contribution has aimed to explore a strategy for biomass valorization through the catalytic conversion of levulinic acid, a platform molecule, to N-heterocycles. A simple procedure has been applied for the synthesis of an active, effective and stable catalytic system with a low noble-metal concentration (g-C_3_N_4_/Pt). The catalytic performance of the aforementioned material was investigated in the continuous flow transformation of levulinic acid to valuable N-heterocycles. A complete parametric analysis was performed by changing the reaction conditions, namely temperature, pressure and concentration of levulinic acid. The optimum balance between conversion and selectivity was found by using 3 M levulinic acid solution in acetonitrile at T = 90°C and P = 50 bars. Remarkably, the catalyst was highly selective (67.5%) to the formation of 1-ethyl-5-methylpyrrolidin-2-one and exceptionally stable during 3 h of reaction.

## Author Contributions

DR-P performed all experiments and wrote the first draft of the manuscript. AP-S supported the experimental work and revised the manuscript draft. AB finalized the draft with RL, conceived the experimental work and provided lab and financial support. MM-B conceived and planned the experiments with RL and finished the manuscript for submission. RL provided the lab for all experiments, planned the experimental work, and finalized and submitted the manuscript.

### Conflict of Interest Statement

The authors declare that the research was conducted in the absence of any commercial or financial relationships that could be construed as a potential conflict of interest. The reviewer CL declared a past co-authorship with one of the authors RL to the handling editor. The author AB had also previously collaborated with the reviewer CL.
