# Correction: Continuous flow synthesis of high valuable N-heterocycles via catalytic conversion of levulinic acid

**DOI:** 10.3389/fchem.2026.1827629

**Published:** 2026-03-23

**Authors:** Frontiers Editorial Office

**Keywords:** N-heterocycles, heterogeneous catalysis, graphitic carbon nitride, continuous flow, platinum, levulinic acid

In the published article, the Conflict of Interest lacked necessary details regarding a previous collaboration between the author Alina M. Balu and the reviewer Christophe Len. An investigation by Frontiers’ Research Integrity auditing team found that this conflict of interest did not unduly impact the peer review process or scientific validity of the article. The correct statement appears below:

“The authors declare that the research was conducted in the absence of any commercial or financial relationships that could be construed as a potential conflict of interest. The reviewer CL declared a past co-authorship with one of the authors RL to the handling editor. The author AB had also previously collaborated with the reviewer CL.”

The original article has been updated.

