# Supplementary material for: Continuous Flow Synthesis of High Valuable N-Heterocycles via Catalytic Conversion of Levulinic Acid
Source: Front Chem. 2019 Feb 26;7:103. doi: 10.3389/fchem.2019.00103 (PMC6399384; doi:10.3389/fchem.2019.00103)
Supplement: Supplementary file 1 [file Table_1.DOCX]

Synthesis of high valuable N-heterocycles through the catalytic conversion of levulinic acid

Daily Rodríguez-Padrón,^[a]^ Alain R. Puente-Santiago,^[a]^ Alina M. Balu^,[a]^ Mario J. Muñoz-Batista,^*[a]^ and Rafael Luque^*[a,b]^

^a^ *Departamento de Química Orgánica, Grupo FQM-383, Universidad de Cordoba, Campus de Rabanales, Edificio Marie Curie (C-3), Ctra Nnal IV-A, Km 396, E14014, Cordoba, Spain*.

*^b^ Peoples Friendship University of Russia (RUDN University), 6 Miklukho-Maklaya street, Moscow, 117198, Russia.*

Rafael Luque: [*q62alsor@uco.es*](mailto:q62alsor@uco.es)*,* Mario J. Muñoz-Batista: [*qo2mubam@uco.es*](mailto:qo2mubam@uco.es) *jmunoz385x@gmail.com*

**Abstract:** Graphitic carbon nitride (g-C_3_N_4_) was successfully functionalized with a low platinum loading to give rise to an effective and stable catalytic material. The synthesized g-C_3_N_4_/Pt was fully characterized by XRD, N_2_ physisorption, XPS, SEM-Mapping and TEM techniques. Remarkably, XPS analysis revealed that Pt was in a dominant metallic state. In addition, XPS together with XRD and N_2_ physisorption measurements indicated that the g-C_3_N_4_ preserves its native structure after the platinum deposition process. g-C_3_N_4_/Pt was applied to the catalytic conversion of levulinic acid to N-heterocycles under continuous flow conditions. Reaction parameters (temperature, pressure and concentration of levulinic acid) were studied using 3 level for each parameter, and the best conditions were applied for the analysis of the catalyst stability. The catalytic system displayed high selectivity to 1‑ethyl-5-methylpyrrolidin-2-one and outstanding stability after 3 h of reaction.

**Keywords:** N-Heterocycles, catalytic reaction, graphitic carbon nitride, continuous flow.

Figure S1. GC-MS spectra of the products obtained from the continuous flow conversion of levulinic acid to Nitrogen-heterocycles. (A) SP1: 1‑ethyl-5-methylpyrrolidin-2-one. (B) SP2: 1-ethyl-2-(ethylideneamino)-5-methylpyrrolidin-2-ol
